# Evaluation of risk factors, clinic pathological aspects and implant characteristics in patients of oral peri-implant malignancies

**DOI:** 10.6026/9732063002001164

**Published:** 2024-09-30

**Authors:** Siddharam M Patil, Piyush Javiya, Gargi Yumnam, Sonal Tripathi, Swati Tripathi, Naina Pattnaik

**Affiliations:** 1 Department of Prosthodontics, PMNM Dental College and Hospital, Navnagar, Bagalkot - 587103, India; 2 Department of Prosthodontics, Crown & Bridge, K. M. Shah Dental College & Hospital, Sumandeep Vidyapeeth Deemed to be University, Piparia, Waghodia, Vadodara, Gujarat, India; 3 Department of Paediatrics and Preventive Dentistry, Dental College, RIMS, Imphal, Manipur, India; 4 Department of Prosthodontics Crown Bridge and Implantology, Rishiraj College of Dental Sciences and Research Centre, Bhopal, Madhya Pradesh, India; 5 Department of Orthodontics, Rishiraj College of Dental Sciences and Research Centre, Bhopal, Madhya Pradesh, India; 6 Department of Periodontics and Oral Implantology, Kalinga Institute of Dental Science, KIIT Deemed to be University, Patia, Bhubaneswar, Odisha, India

**Keywords:** Oral peri-implant malignancies, retrospective analysis

## Abstract

This study was conducted to carry out retrospective analyses of oral peri-implant malignancy (OPIM) focussing on demographic details, risk factors, habit factors, clinicopathological features and implant features in patient with OPIM. Clinical data and demographic data from 1646 individuals with oral cancer undergoing resection procedures were gathered. Clinical, radiological, histopathological assessments and implant characteristics records of these patients were obtained and assessed. 46 (2.79%) cases were found to diagnose with OPIM. 36 (85.76%) cases of OPIM were found to having implant occlusion with opposing prosthesis. Prosthesis/prosthesis occlusion was observed in 18 (50%) cases, prosthesis/inlay occlusion in 4 (11.12%) cases and prosthesis/prosthesis on implants in 14 (38.88%) cases. The surgical placement of implants and the galvanic currents that flow between prostheses can operate as an irritating or inflammatory cofactor that aids in the development of malignancies.

## Background:

One of the most significant forms of treatment available to people who are either completely or partially edentulous is oral rehabilitation through dental implant [1-3]. Peri-implantitis (PI), an inflammatory condition of the alveolar bone and surrounding soft tissues of dental implants, has become more common as a result of the widespread application of dental implants [3 -5]. Gingival ulcers, gingival hypertrophy, gingival hyperplasia, gingival edematous swelling with erythema is the clinical manifestations of PI [6-8]. Surgical biopsies may occasionally be necessary for the differential identification of malignant tumors in these presentations [9-12]. Oral cavity malignancies account for anywhere from three to five percent of all human malignancies or approximately fifty percent of all cancers of the head and neck [12-14].Oral squamous cell carcinomas (OSCCs) account for more than ninety percent of malignancies of oral cavity [15-18]. Their multifaceted etiology includes tobacco and alcohol addiction, males more than 60 years of age, exposure to sunlight, infections with HPV, diets high in fat, nutritional deficiencies [19-21]. There have been numerous modifications causing occurrence of OSCC in people less than forty years of age including adolescents, children and women who don't have any known risk factors [22-24]. Other less common risk factors that have also been recognized include immunosuppressive medications, chronic inflammation in conjunction with periodontitis and multiple irritating factors related to the origin of teeth and/or dental implants [11-15]. As more implants are inserted, there are more incidences of OSCC involving implants [13-17]. Several papers addressing osseointegrated dental implants related to SCC cases have been released to date, albeit under various titles [3-9]. Therefore, we coined the phrase "oral peri-implant malignancy" (OPIM) to refer to all types of cancers that accompany dental implants. The present study was conducted to carry out retrospective analyses of OPIM focussing on risk factors, habit factors, clinicopathological features and implant features in patient with OPIM.

## Methods and Materials:

## Obtaining patient data:

Clinical data and demographic data from 1646 individuals with oral cancer undergoing resection procedures were gathered. Patients with oral cancer between May 2016 and May 2014 were included in this study.

## Qualifications for inclusion:

Patients with malignant tumor mass had dental implant.

## Criteria for exclusion:

This study excluded patients with dental implants next to malignant primary masses. On the basis of exclusion and inclusion criteria, 42 patients were selected in which dental implant was present inside the malignant tumor mass.

## Clinical examination:

Chart assessments, radiographs and clinical pictures were used to gather clinical and radiographic information. Assessments were conducted on the following topics: fundamental demographic information, location of malignancy, clinical characteristics of malignancy, location of implant, cancer progression period after placement of dental implant, type of prosthesis and surface of implants, bone graft methods, the existence of precancerous lesions and conditions, risk variables like drinking alcohol and tobacco consumption and maintenance of oral hygiene. The peri-implant image or panorama was used to calculate the marginal bone loss.

## Histopathologic analysis:

All histological analyses were carried out at Department of Oral Pathology. A 5-10 mm specimen was taken from the middle of the primary main mass for HPV testing after the primary main tissue was acquired in the operating room. The pathological reports were evaluated to gather information on the pathologic diagnosis, the site of the primary tumor, the pathologic staging according to the eighth edition of the American Joint Committee on Cancer (AJCC) staging system, the degree of differentiation, and the presence of bone involvement.

## Statistical analysis:

The data collected were put in MS excel sheet and put for statistical analysis. Data was represented in the form of percentages. Chi square test was used for statistical analysis. P value ≤ 0.05 was taken as statistically significant. SPSS version 21 was used for statistical analysis.

## Results:

In this study details of 1646 patients who underwent surgery for oral malignancy were evaluated. 46 (2.79%) cases were found to diagnose with OPIM. Average duration between diagnosis of OPIM and implant insertion was 48.24 ± 34.74 months (Table 1).

The OPIM was more common in males (61.9%) as compared to female (38.1%). The most common age group affected were 50-59 years (28.57%) and 60-69 years (38.09%). Mandible was more commonly affected (66.64%) than maxilla (33.34%) The findings were significant statistically (p <0.001) (Table 2).

All cases were previously treated for peri-implantitis. Most of the patients were found to have moderate oral hygiene (71.42%) followed by poor oral hygiene (23.80%).4 (9.52%) patients with OPIM were found to have history of smoking tobacco, 3 (7.14%) patients had habit of chewing tobacco, 4 (9.52%) patients were found to have addiction for alcohol while 10 (23.80%) patients were found to have history of more than one habit. 18 (42.85%) patients were HPV positive (Table 3).

The clinical appearance of lesions was exophytic in nature in 18 (42.85%) patients, while it was ulcerative in 4 (9.52%) patients and exophytic -ulcerative in 20 (47.61) patients with OPIM .40 (95.23%) patients were histopathologically diagnosed with oral squamous cell carcinoma (OSCC). 2 (4.77%) were diagnosed with oral melanoma. The findings were significant statistically (p <0.001). Histopathological staging of 30 (75) of the peri-implant OSCC was 4. Most of the cases were in histopathological staging 4. 6 (15.0%) were in stage 2 while 4 (10%) were in stage 1. The findings were significant statistically. (p <0.001).Most of the peri-implant OSCC were well differentiated (80%). While 4 (10%) each were moderately differentiated and poorly differentiated. The findings were significant statistically. (p<0.001).It was observed that 30 (75%) of peri-implant OSCC were found to involve bone. The findings were significant statistically (p <0.001) (Table 4).

Bone grafts were used in implant placement in 22 patients of peri-implant oral malignancy. Autogenous bone grafts was used in 6 (27.27%) cases, allogeneic bone grafts in 2 (9.09%) cases, xenogeneic bone grafts in 12 (54.54%) cases and synthetic bone graft in 2 (9.09%) cases. Different type of surface treatment was performed over implants like Ti Unite, Sandblast and acid etching, HA-coated, Titanium plasma spray, TiO2. 36 cases were found to having implant occlusion with opposing prosthesis. Prosthesis/prosthesis occlusion was observed in 18 (50%) cases, prosthesis/inlay occlusion in 4 (11.12%) cases and prosthesis/prosthesis on implants in 14 (38.88%) cases (Table 5).

## Discussion:

Despite the fact that dental implants had great clinical success in recent years, there are some concerning reports in the literature that link OSCC to dental implants [11-19]. These findings of our research are similar to the findings of a research which also found around 3% cases of OPIM among the cases of oral malignancies evaluated over duration of five years [19-26]. Literature showed that more number of cases was found in OPIM male patients as compared to females. It was found that most common age group of patients with OPIM is between 60-70 years of age [20-24]. The present research also found that most common age group is 60-69 years. Like present research, another research also found that mandible is most common site of OPIM as compared to maxilla [23- 26].

The findings of the present study have similarity with other literature that showed peri-implantitis to be a regular feature in cases of OPIM [20-23]. Besides, other studies stated that tobacco chewing, tobacco smoking and drinking alcohol was observed in about one fifth of cases of OPIM [21-26]. It has been found in literature that HPV was positive around 45% of OPIM [18-20].

There is a research that has shown that most common clinical appearance of OPIM is exophytic -ulcerative growth [16-20]. The finding is similar to finding of present research. In other studies, the most common type of OPIM reported is OSCC, with most cases being well-differentiated and classified as stage 4. These findings are consistent with those observed in our current research. [20- 26].

There are several multiple case report along with associated review articles like our research has shown that potential contributing variables of relevant OPIM could be (1) corrosion of dental implants and potential correlation between products of corrosion and OSCC [13-16]; (2) potential correlation between particulate matter of titanium and OSCC[14-18]; (3) movement of cancerous cells by means of the sulcus surrounding implant[15- 19]; and (4) potential carcinogenic consequence of sustained metallic ion discharge after the placement of implants [7-13]. The degradation of pure titanium alloy from the attachments to the adjacent media may result in electrochemical or galvanic corrosion, which could be linked to the release of corrosion products and OSCC [8-12]. This theory might hold water, particularly in the case of malfunctioning or failing implants, which happen frequently in PI cases, with a corrosion rate of just 0.003 µA/cm2, titanium ions are among the most stable metallic ions [15- 18]. Inflammation surrounding orthopedic implants may be caused by particulate implant debris. Consequently, implant bursitis and bone resorption may result from inflammatory agents [8- 14]. Malignant cells can enter by means of dental implants, and it has been proposed that PIOM is caused by the passage of cells across implants that come into touch with the gingival sulcus [11- 16]. Based on three theories, a more thorough analysis of the carcinogenic impact of metallic ion discharge has been proposed [18-20]. This analysis is broken down into three distinct issues: (1) the potential of carcinoma by the metallic ions; (2) the individual's exposure degree; and (3) the frequency of malignancies in patients who received implants [21 -24]. In addition to titanium ions' corrosive carcinogenicity, the patient's exposure amount will rely on the implant's outermost area and length of exposure [25, 26]. The present research like some other research observed that implants as well as galvanic currents between various prostheses may be irritants and/or inflammatory cofactors that contribute to the onset and/or progression of OSCC [20 -26]. To clearly establish a cause-effect relationship, basic research is required. Although the frequency of carcinomas adjacent to dental implants is minimal, as dental implant therapy increases, it may become clinically significant [1-20]. Careful assessments and customized recall intervals may be beneficial for patients who are at risk. Prior to implant treatment, a careful evaluation of the oral cavity is necessary, and the patient's SCC risk factors need to be closely monitored and controlled using stringent follow-up procedures [1-26]. For patients who have risk factors, appropriate routine tests should be carried out and any suspicious lesions should prompt the completion of a histopathologic biopsy investigation [1, 26].

## Conclusion:

The surgical placement of implants and the galvanic currents that flow between prostheses can operate as an irritating or inflammatory cofactor that aids in the development of malignancies. Individualized recall intervals and thorough examinations may be beneficial for patients who are at risk.

## Figures and Tables

**Table 1:** Details of patient of oral peri-implant malignancy

**Total number of operated oral cancer patients evaluated**	**1646**
Number of patients with OPIM	46 (2.79%)
Average duration between diagnosis of OPIM and implant insertion	48.24 ± 34.74 months

**Table 2:** Data showing details of demographic features of patients with OPIM

		**N (%)**	**P value**
Gender	Male	26 (61.9)	<0.001*
	Female	16 (38.1)	
Age (years)	40-49	6 (14.28)	
	50-59	12 (28.57)	<0.001*
	60-69	16 (38.09)	
	70-79	4 (9.52)	
Site	Maxilla	14 (33.34)	<0.001*
	Mandible	28 (66.64)	

**Table 3:** Data showing details of risk factors and habitual factors in patients with OPIM

		**N (%)**
Oral hygiene	Poor	10 (23.80)
	Moderate	30 (71.42)
	Good	2 (4.77)
Habits	Smoking tobacco	4 (9.52%)
	Chewing tobacco	3 (7.14%)
	Drinking alcohol	4 (9.52%)
	More than one habit	10 (23.80%)
HPV	Positive	18 (42.85%)
	Negative	24 (57.14%)

**Table 4:** Data about clinic-histopathological details of patients with OPIM

**Clinical features (n=42)**	**N (%)**	**P value**
Exophytic	18 (42.85)	
Ulcerative	4 (9.52)	0.876
Exophytic-ulcerative	20 (47.61)	
Diagnosis (n=42)		
Oral Squamous cell carcinoma (OSCC)	40 (95.23)	<0.001*
Oral Melanoma	2 (4.77)	
Histopathological staging (n=40)		
1	4 (10.0)	
2	6 (15.0)	<0.001*
4	30 (75)	
Histopathological features (n=40)		
Well differentiated	32 (80%)	
Moderate differentiated	4 (10%)	<0.001*
Poorly differentiated	4 (10%)	
Bone involvement (n=40)		P value
Yes	30 (75)	<0.001*
No	10 (25)	

**Table 5:** Details about the implants in patients with peri-implant oral malignancy

**Bone graft (n=22)**	**Autogenous bone grafts**	**6 (27.27%)**
	Allogenic bone grafts	2 (9.09%)
	Xenogenic bone grafts	12 (54.54%)
	Synthetic bone grafts	2 (9.09%)
Surface treatment of implants (n=42)	Ti Unite	8 (19.04%)
	Sandblast and acid etching	12 (28.57%)
	HA-coated	4 (9.52%)
	Resorbable blast media	8 (19.04%)
	Titanium plasma spray	2 (4.76%)
	Hydroxyapatite blast and acid wash	4 (9.52%)
	TiO2	4 (9.52%)
Opposing occlusion pros theses (n=36)	Prosthesis/prosthesis occlusion	18 (50%)
	Prosthesis/inlay occlusion	4 (11.12%)
	Prosthesis/prosthesis on implants	14 (38.88%)
